# Estimating surgery, radiotherapy and systemic anti-cancer therapy treatment costs for cancer patients by stage at diagnosis

**DOI:** 10.1007/s10198-023-01623-5

**Published:** 2023-09-01

**Authors:** Lorna Wills, Diana Nagarwalla, Clare Pearson, Sean McPhail, Rose Hinchliffe, Ben Sharpless, Fahmina Fardus-Reid, Lyndsy Ambler, Samantha Harrison, Jon Shelton

**Affiliations:** 1 Cancer Research UK, 2 Redman Place, London, E20 1JQ UK; 2 National Cancer Registration and Analysis Service, NHS Digital, 10 South Colonnade, London, E14 4PU UK

**Keywords:** Cancer, Treatment, Costs, Stage at diagnosis, H51

## Abstract

**Background:**

The increasing burden of cancer has economic implications for the healthcare system in England. However, there is limited evidence on the cost of cancer treatment. We calculated the costs of initial cancer treatment (resection, radiotherapy, systemic anti-cancer therapy [SACT]) based on stage at diagnosis.

**Methods:**

Data from England’s National Cancer Registration Dataset were matched to English Hospital, Radiotherapy and SACT data for breast, lung, prostate, colon and rectal cancers diagnosed between 2016 and 2018. Treatment data were matched to National Schedule of Reference Costs data to calculate the cost of each treatment event.

**Results:**

Breast, colon and rectal cancers treated with resection, radiotherapy or SACT had increasing costs with later stage at diagnosis; costs for lung and prostate cancers were lower at stages 1 and 4 compared to stages 2 and 3. In general, surgery and SACT were the most expensive treatments. Radiotherapy and SACT costs showed little change across stages 1–3; radiotherapy costs decreased in stage 4, while SACT costs increased.

**Conclusions:**

This analysis estimates initial treatment costs by stage based on observed data. Future research can build on this to provide more comprehensive costings associated with cancer; this is important for future planning of cancer services.

**Supplementary Information:**

The online version contains supplementary material available at 10.1007/s10198-023-01623-5.

## Background

Cancer is a leading cause of morbidity and mortality in the United Kingdom (UK) [1] and accounts for a considerable proportion of the annual expenditure of the National Health Service (NHS) [2]. There are approximately 375,000 cases of cancer diagnosed each year in the UK [3] and more than 166,000 deaths due to cancer [4]. Overall cancer incidence and mortality are projected to increase substantially in the future [5], largely driven by an aging and increasing population. This has both resource and cost implications for the NHS. An estimate of the cost of cancer services for the NHS in England was approximately £6.7 billion per year in 2012/2013, expected to grow 9% each year [6]; however, there is a lack of recent data on the cost of cancer services or treatment.

Early diagnosis is a key component of initiatives to improve cancer outcomes, with the NHS Long Term Plan in England setting an ambition to increase the proportion of stageable cancer patients diagnosed at stage 1 and 2 to 75% by 2028 [7]. Patients diagnosed at earlier stages are often more likely to receive curative treatment and have better outcomes than those diagnosed at later stages [8–10]. Early diagnosis has also been highlighted as a way to reduce the longer-term burden on the healthcare system [11]; however, the impact of diagnosing patients at an earlier stage on the total cost of care to the health system is not as well established as the benefits on cancer outcomes and quality of life for patients. This paper adds to the literature by focusing on the costs of the three main treatment modalities (resective surgery, radiotherapy or systemic anti-cancer therapy [SACT]) for the cancer sites of interest.

Previous work on costs of cancer care using English cancer registry data either focused on a particular cancer site (i.e., skin [12] or breast cancer [13]) and/or used relatively old cancer registration data (2008 [12]; 2001–2010 [14]) in which poor completeness of stage at diagnosis data limits the analysis of treatment costs by stage. Other research has estimated costs of cancer [15, 16] but these estimates have been based on modelled treatment pathways rather than observed treatment-related data at an individual patient level. Where these previous studies included an analysis of cost by stage, they generally found that cost of cancer increased with later stage at diagnosis. Data on SACT has routinely been collected from NHS trusts since 2014 [17] and recent improvements in the completeness of staging data allow for a fuller investigation of costs by stage [18]; a more up to date assessment of treatment costs is beneficial given the rapidly changing treatment landscape, including the introduction of targeted therapies for certain cancers.

Calculating the cost of treatment from administrative datasets is methodologically complex, with a recent systematic review highlighting several methodological issues with previous cost estimation research, such as how to exclude treatment not related to the diagnosis of interest and issues surrounding missing data [19]. Using methodology developed to determine whether a patient received resective surgery, radiotherapy or SACT within an initial treatment timeframe [20], the current study builds on this to calculate the costs of these initial treatments in England by stage at diagnosis, focusing on five cancer sites (breast, lung, prostate, colon and rectal). Estimating initial treatment costs by stage at diagnosis will inform discussions on the impact of early diagnosis initiatives from an economic perspective, such as the economic implications of meeting the NHS Long Term Plan ambition [7].

## Methods

### Cohort identification

This study used the English National Cancer Registration Dataset collected by the National Cancer Registration and Analysis Service [21]. As patients can have multiple tumours at the same or different sites, analysis was undertaken using data at tumour rather than patient level. The study population included breast (ICD10 code C50), colon (C18 and C19), rectal (C20), lung (C33 and C34) and prostate (C61) tumours diagnosed in individuals over the age of 18 between 2016 and 2018 in England. These cancers were chosen as they have the highest incidence in the UK and demonstrate a range of treatment patterns across the different cancer sites and by stage. Separate analyses were undertaken for colon and rectal tumours as treatment guidelines are different for these sites [22]; however, results are also presented for colorectal cancer together in view of early diagnosis initiatives tending to target combined colorectal cancers.

The following records were excluded: breast tumours diagnosed in male patients or diagnosed at stage 0 due to small numbers; tumours treated within a SACT-based clinical trial due to the unknown costs associated with this; and any tumours where the patient had another cancer diagnosis except for non-melanoma skin cancer (ICD10 code C00-C97 excluding C44) within 18 months of the diagnosis of interest to reduce the risk of attributing a given treatment to the incorrect diagnosis.

Cancer stage at diagnosis was classified using the TNM system as stage 1–4. Patient characteristics – age, gender, ethnicity, deprivation, number of comorbidities and stage at diagnosis – were extracted. The Charlson Comorbidity Index [23] for a patient was derived using diagnostic codes in the Hospital Episode Statistics (HES) dataset and National Cancer Registration Dataset in the period between 27 and 3 months before diagnosis. Deprivation quintile was derived using the income domain of the Indices of Multiple Deprivation [24].

### Treatment data

Three treatment modalities were included with data extracted from three datasets: resective surgery (“resection”; Admitted Patient Care Hospital Episode Statistics) [25], radiotherapy (Radiotherapy Dataset [RTDS]) [26] and SACT (Systemic Anti-Cancer Therapy dataset) [27]. SACT includes chemotherapy, targeted therapy, immunotherapy and endocrine therapy delivered in secondary or tertiary care settings [17]. Any endocrine therapies administered alone and not in combination with another treatment of interest were excluded.

Timeframes for initial treatment for each cancer and treatment modality were based on previous work that used a data-driven approach supplemented by clinical guidance to identify a period within which the primary course of treatment was likely to be captured but which minimised the inclusion of treatment for recurrence [20]. For each cancer, the number of days after diagnosis at which 95% of patients received treatment was identified and then rounded to the nearest three-month interval. Initial treatment timeframes included the 31 days prior to diagnosis and six to 15 months post-diagnosis, depending on cancer site and treatment modality (Online Resource 1). Cancer- and, where appropriate, stage-specific Operating Procedure Codes Supplement (OPCS) for resection of primary tumour were taken from previous research [20].

### Costing data

Costs were assigned to each treatment using the 2017/2018 National Schedule of Reference Costs [28]. Reference costs give the mean unit cost of providing a defined service (classified using Healthcare Resource Group [HRG] codes) to NHS patients in England for the year in question. These reference costs are stratified by the setting in which care was delivered (inpatient elective, day case, outpatient, etc.). The average cost across all settings was used for each treatment type, as it was not possible to identify the setting from the treatment datasets used.

To match treatment events to the reference costs data, HRG codes for each treatment event were identified, where possible. When it was not possible to directly identify HRG codes, costs were assigned in a hierarchical way (see Online Resource 1). Additional costs, including diagnostic tests, hospital admissions, critical care unit beds and supportive medicines, were not included.

### Calculating cost per tumour

For each individual tumour, the cost of primary resection, radiotherapy or SACT was calculated as the sum of the costs of each individual treatment event (Online Resource 1). We also calculated an overall treatment cost for each tumour, as the sum of the total cost of resection, total cost of radiotherapy and total cost of SACT received by a tumour. Tumours that either had no treatment recorded, or where the treatment recorded could not be costed, were assigned a cost of £0. The mean cost of treatment was calculated for all tumours that had a costed treatment event for the modality of interest (“treated tumours”) as well as for all tumours in the cohort (“all tumours”) regardless of whether they received costed treatment or not. We also calculated the mean cost of treatment stratified by patient characteristics. Due to the inherent uncertainty in these estimates, confidence intervals were not calculated and comparisons between mean costs are descriptive rather than based on statistical significance.

### Sensitivity analysis

A sensitivity analysis for assigning costs to SACT was carried out due to the uncertainty around the costing process. For this sensitivity analysis we assigned the cost for procuring and delivering regimens that do not appear on the National Tariff Chemotherapy Regimens List to all cycles where it was not possible to directly assign an OPCS/HRG code to an individual cycle of treatment (further details in Online Resource 1).

## Results

### Cohort demographics

The final analysis included 455,789 tumours, of which 125,294 were breast tumours, 108,602 were lung, 125,609 were prostate, 69,713 were colon, and 26,571 were rectal tumours. Patient characteristics by cancer site are shown in Table 1.

**Table 1:** Patient characteristics by site

		Breast (*N* (%))	Lung (*N* (%))	Prostate (*N* (%))	Colon (*N* (%))	Rectal (*N* (%))
Total		125,294	108,602	125,609	69,713	26,571
Gender	Female	125,294 (100)	51,596 (47.5)	0 (0.0)	33,454 (48.0)	9,668 (36.4)
	Male	0 (0.0)	57,006 (52.5)	125,609 (100)	36,259 (52.0)	16,903 (63.6)
Age at diagnosis	< 45	11,646 (9.3)	1,002 (0.9)	246 (0.2)	2,537 (3.6)	1,104 (4.2)
	45–54	26,505 (21.2)	5,101 (4.7)	5,579 (4.4)	4,512 (6.5)	2,411 (9.1)
	55–64	27,900 (22.3)	16,996 (15.6)	25,508 (20.3)	11,175 (16.0)	5,803 (21.8)
	65–74	30,235 (24.1)	37,107 (34.2)	51,983 (41.4)	19,046 (27.3)	7,994 (30.1)
	75–84	18,836 (15.0)	34,173 (31.5)	32,544 (25.9)	21,024 (30.2)	6,406 (24.1)
	85 +	10,172 (8.1)	14,223 (13.1)	9,749 (7.8)	11,419 (16.4)	2,853 (10.7)
Stage at diagnosis	1	51,801 (41.3)	18,985 (17.5)	41,314 (32.9)	9,536 (13.7)	6,184 (23.3)
	2	48,351 (38.6)	7,876 (7.3)	19,754 (15.7)	17,435 (25.0)	4,501 (16.9)
	3	10,548 (8.4)	21,286 (19.6)	28,135 (22.4)	17,285 (24.8)	8,779 (33.0)
	4	6,090 (4.9)	52,803 (48.6)	23,688 (18.9)	17,935 (25.7)	4,964 (18.7)
	Unknown	8,504 (6.8)	7,652 (7.0)	12,718 (10.1)	7,522 (10.8)	2,143 (8.1)
Ethnicity	Asian	4,753 (3.8)	1,885 (1.7)	2,374 (1.9)	1,467 (2.1)	742 (2.8)
	Black	2,650 (2.1)	1,143 (1.1)	4,292 (3.4)	1,142 (1.6)	320 (1.2)
	White	108,485 (86.6)	99,742 (91.8)	108,385 (86.3)	62,552 (89.7)	23,762 (89.4)
	Mixed	703 (0.6)	302 (0.3)	539 (0.4)	239 (0.3)	99 (0.4)
	Not stated or known	6,705 (5.4)	4,499 (4.1)	8,689 (6.9)	3,570 (5.1)	1,359 (5.1)
	Other	1,998 (1.6)	1,031 (0.9)	1,330 (1.1)	743 (1.1)	289 (1.1)
Deprivation quintile	1 – Least deprived	28,993 (23.1)	15,525 (14.3)	31,760 (25.3)	15,487 (22.2)	5,900 (22.2)
	2	28,743 (22.9)	19,677 (18.1)	30,728 (24.5)	15,974 (22.9)	5,957 (22.4)
	3	25,944 (20.7)	21,617 (19.9)	25,909 (20.6)	14,432 (20.7)	5,663 (21.3)
	4	22,570 (18.0)	24,069 (22.2)	21,108 (16.8)	12,630 (18.1)	4,858 (18.3)
	5 – Most deprived	19,044 (15.2)	27,714 (25.5)	16,104 (12.8)	11,190 (16.1)	4,193 (15.8)
Charlson comorbidity score	0	108,918 (86.9)	73,382 (67.6)	103,708 (82.6)	54,214 (77.8)	21,976 (82.7)
	1	9,176 (7.3)	15,737 (14.5)	11,984 (9.5)	7,413 (10.6)	2,340 (8.8)
	2	3,797 (3.0)	9,194 (8.5)	5,270 (4.2)	3,926 (5.6)	1,157 (4.4)
	3 +	3,403 (2.7)	10,289 (9.5)	4,647 (3.7)	4,160 (6.0)	1,098 (4.1)

There was a record of receiving costed treatment in 84.8% of breast tumours, 54.3% of lung tumours, 52.1% of prostate tumours, 71.6% of colon tumours, and 83.9% of rectal tumours. Overall, 66.4% of tumours received costed treatment. Proportions receiving treatment by site and stage at diagnosis are presented in Online Resource 2.

### Resection costs

#### Analysis among cohort of tumours that received resection

**Fig. 1:**
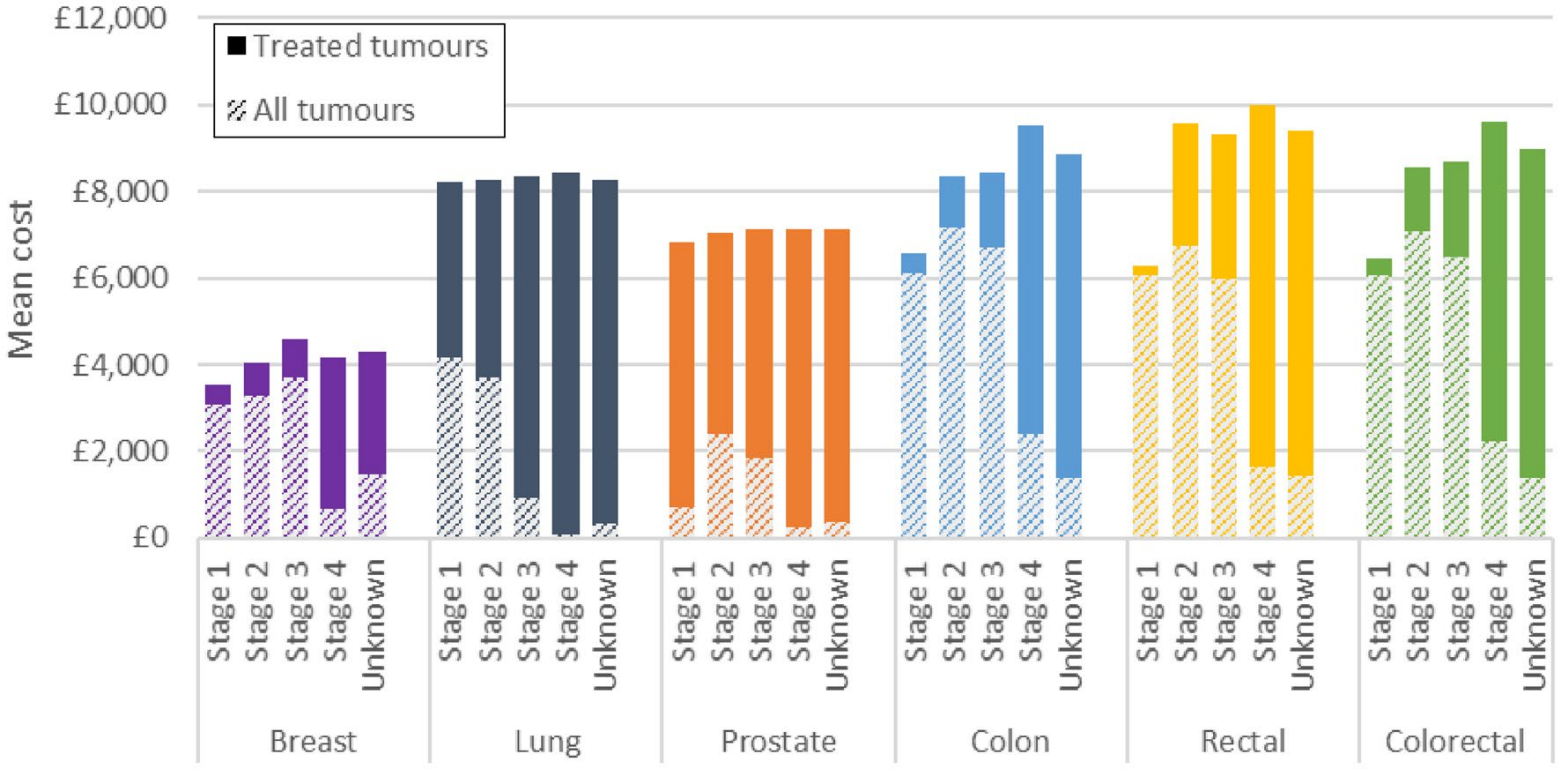
Mean cost of resection by cancer and stage at diagnosis. Solid bars show the incremental mean cost of resection for treated tumours compared to the mean cost for all tumours. Proportions of patients receiving resection were 77% (breast); 15% (lung); 16% (prostate); 62% (colon); 60% (rectal); 62% (colorectal). The proportion of patients receiving treatment varied by stage at diagnosis. Treated tumours refers to tumours treated with resection (alone or in combination with another modality). All tumours refers to all tumours in the analysis cohort regardless of whether they received costed treatment or not

The mean cost of resection for treated tumours was £3,876 for breast cancer, £8,252 for lung cancer, £7,024 for prostate cancer, £8,149 for colon cancer and £8,261 for rectal cancer. Colon and rectal cancers combined had a mean cost of £8,179. The mean cost was similar across all stages for lung and prostate cancers, while breast, colon and rectal cancers had lower costs at stage 1 than at later stages (Fig. 1). The costs for breast cancer were lower than the other cancers at each stage. The lower costs for stage 1 colon and rectal cancers is likely due to the inclusion of less costly endoscopic procedures within the resection cohort at stage 1, while the lower cost for stage 1 breast cancer may be driven by lower complexity of surgery at earlier stages, with different costs assigned to the same procedure based on different ‘complexity levels’ in the reference costs data.

#### Analysis among full tumour cohort

When the analysis was widened to include all tumours in the cohort, the pattern of costs changed due to differing proportions of patients receiving resection. The mean cost of resection for all tumours was £2,976 for breast cancer, £1,245 for lung cancer, £1,106 for prostate cancer, £5,061 for colon cancer and £4,956 for rectal cancer. Colon and rectal cancers combined had a mean cost of £5,032. The relationship between stage and mean cost of resection for all tumours exhibited more variation by cancer site than when only treated tumours were included. For breast, colon and rectal cancers, the cost for stages 1–3 was relatively similar with a large decrease in costs for stage 4 due to the small proportion of patients who received resection at this stage. For lung cancer, costs decreased with later stage at diagnosis, while prostate cancer had lower costs for stages 1 and 4. Across all cancers, the mean cost of resection was highest for colon and rectal cancer at each stage.

### Radiotherapy costs

#### Analysis among cohort of tumours that received radiotherapy

**Fig. 2:**
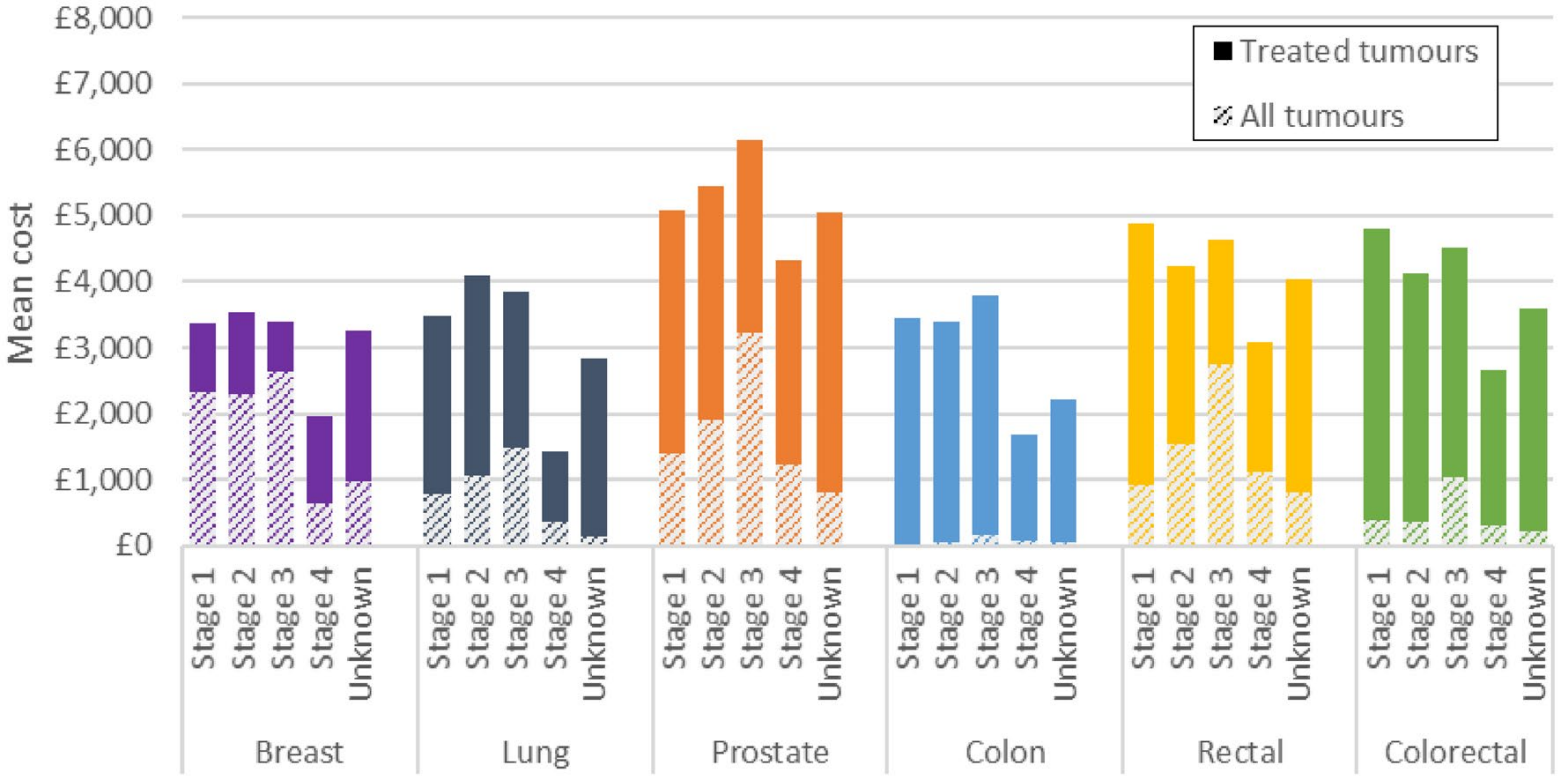
Mean cost of radiotherapy by cancer and stage at diagnosis. Solid bars show the incremental mean cost of radiotherapy for treated tumours compared to the mean cost for all tumours. Proportions of patients receiving radiotherapy were 64% (breast); 26% (lung); 33% (prostate); 3% (colon); 39% (rectal); 13% (colorectal). The proportion of patients receiving treatment varied by stage at diagnosis. Treated tumours refers to tumours treated with radiotherapy (alone or in combination with another modality). All tumours refers to all tumours in the analysis cohort regardless of whether they received costed treatment or not

The mean cost of radiotherapy for treated tumours was £3,393 for breast cancer, £2,655 for lung cancer, £5,390 for prostate cancer, £2,784 for colon cancer and £4,296 for rectal cancer. Colon and rectal cancers combined had a mean cost of £4,047. In general, the cost of radiotherapy among treated tumours was similar for stages 1–3 but lower for stage 4 (Fig. 2). Prostate cancer had a gradient of slightly increasing costs from stages 1 to 3.

#### Analysis among full tumour cohort

The mean cost of radiotherapy for all tumours in the cohort was £2,163 for breast cancer, £692 for lung cancer, £1,792 for prostate cancer, £81 for colon cancer and £1,660 for rectal cancer. Colon and rectal cancers combined had a mean cost of £517. The mean cost of radiotherapy among all tumours showed a similar pattern by stage for lung, prostate, colon and rectal cancers with an increase in mean cost from stages 1–3 and a decrease for tumours diagnosed at stage 4. Breast, however, showed a different pattern with costs similar for stages 1–3 and much lower for stage 4. For each stage, the costs for colon cancer were much lower than other cancer sites, as only 3% of colon cancers were treated with radiotherapy.

### Systemic anti-cancer therapy costs

#### Analysis among cohort of tumours that received SACT

**Fig. 3:**
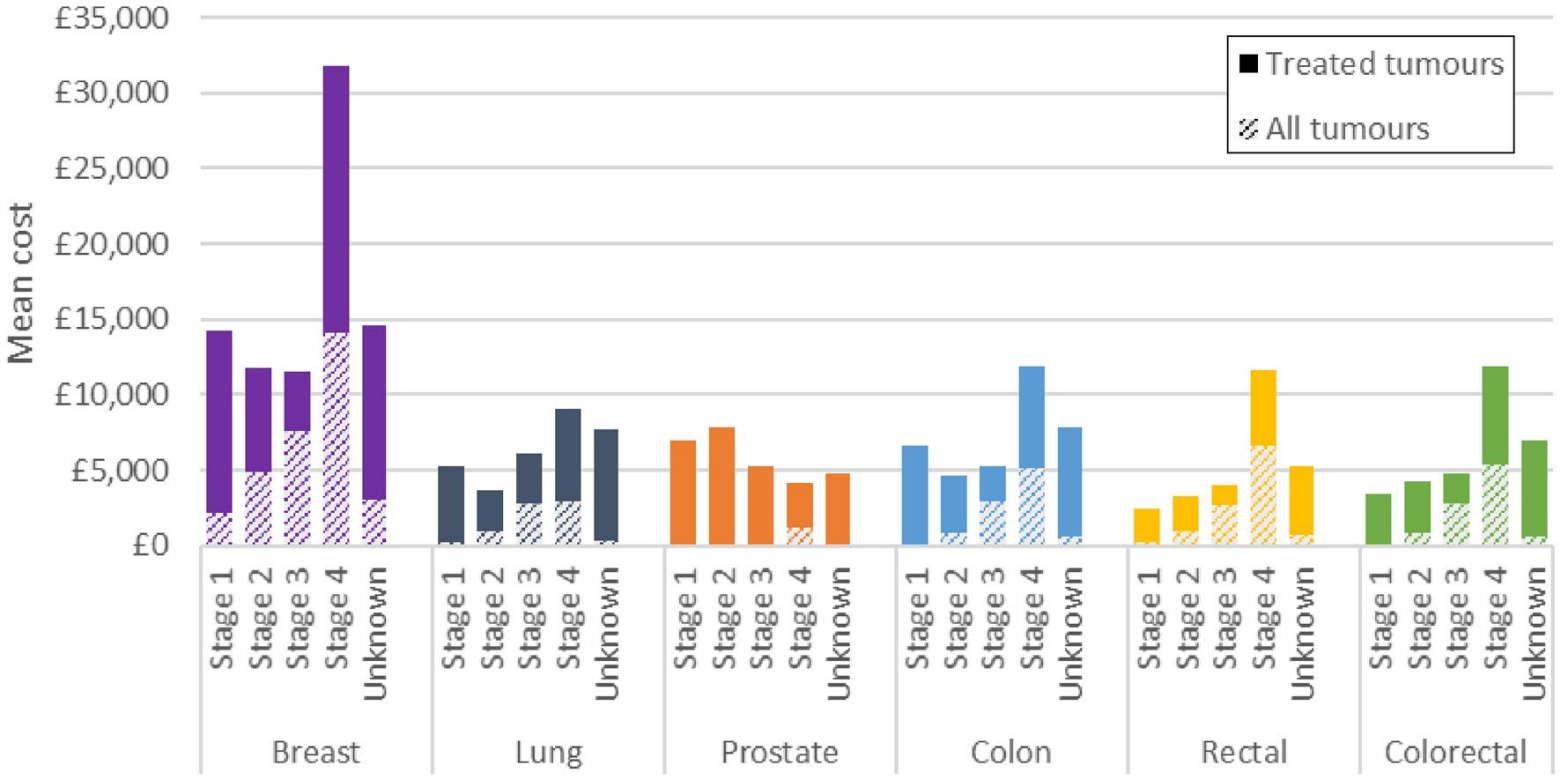
Mean cost of systemic anti-cancer therapy by cancer and stage at diagnosis. Solid bars show the incremental mean cost of systemic anti-cancer therapy for treated tumours compared to the mean cost for all tumours. Proportions of patients receiving systemic anti-cancer therapy were 31% (breast); 28% (lung); 6% (prostate); 30% (colon); 40% (rectal); 33% (colorectal). The proportion of patients receiving treatment varied by stage at diagnosis. Treated tumours refers to tumours treated with SACT (alone or in combination with another modality). All tumours refers to all tumours in the analysis cohort regardless of whether they received costed treatment or not

The mean cost of SACT for treated tumours was £13,696 for breast cancer, £7,670 for lung cancer, £4,350 for prostate cancer, £7,673 for colon cancer and £5,914 for rectal cancer. Colon and rectal cancers combined had a mean cost of £7,078. Across most cancer sites, costs were much higher for stage 4 compared to earlier stages, except for prostate cancer, where costs generally decreased with later stage at diagnosis (Fig. 3). Three cancer sites (breast, lung, colon) had higher costs at stage 1 compared to stage 2.

#### Analysis among full tumour cohort

The mean cost of SACT for all tumours in the cohort was £4,312 for breast cancer, £2,116 for lung cancer, £268 for prostate cancer, £2,296 for colon cancer and £2,372 for rectal cancer. Colon and rectal cancers combined had a mean cost of £2,317. The mean cost of SACT increased with later stage at diagnosis for each cancer, although costs remained similar between stages 3 and 4 for lung cancer. For each stage, the cost was highest for breast cancer and lowest for prostate cancer.

#### Sensitivity analysis

When the reference cost for procuring and delivering regimens not on the National Tariff Chemotherapy Regimens List was used for all cycles without an OPCS code instead of first using the mean cost for a regimen, there were slight changes (< 10%) in the mean cost of SACT but the relationship between stage and mean cost remained unaltered.

### Total costs

**Fig. 4:**
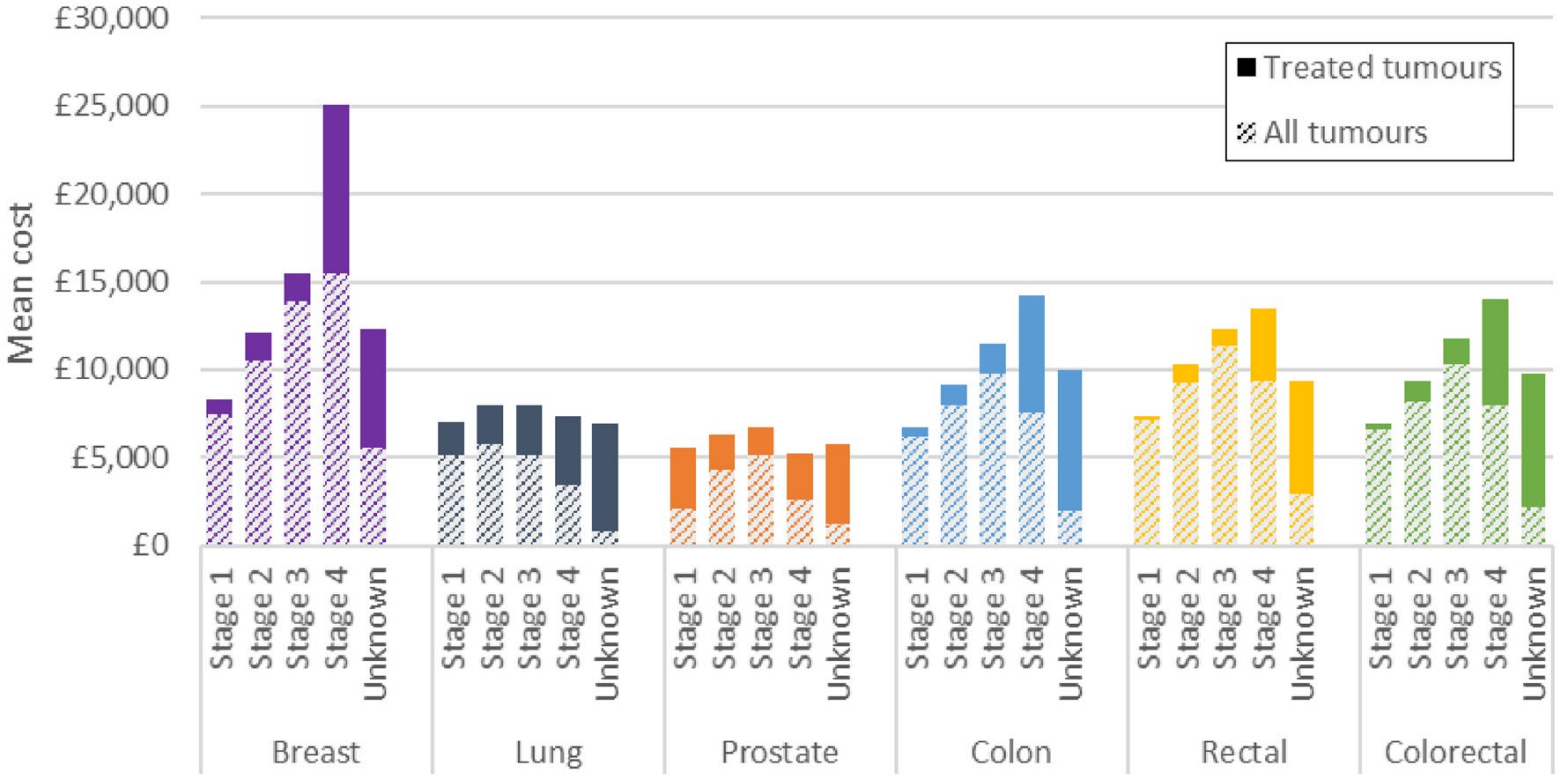
Mean cost of treatment by cancer and stage at diagnosis. Solid bars show the incremental mean cost of treatment for treated tumours compared to the mean cost for all tumours. Treated tumours refers to tumours treated with at least one treatment modality of interest (alone or in combination with another modality). All tumours refers to all tumours in the analysis cohort regardless of whether they received costed treatment or not

Among tumours treated with at least one modality of interest, the mean cost increased with later stage at diagnosis for breast, colon and rectal cancers and showed a slight inverse U-shaped relationship for lung and prostate cancer with lower costs at stages 1 and 4 (Fig. 4). The mean total cost among the full tumour cohort increased with later stage at diagnosis for breast cancer but showed an inverse U-shaped relationship for all other cancers with lower costs at stages 1 and 4. The cost of treatment at stage 4 was higher than the cost at stage 1 for breast, prostate, colon and rectal cancers but lower than at stage 1 for lung cancer.

**Fig. 5:**
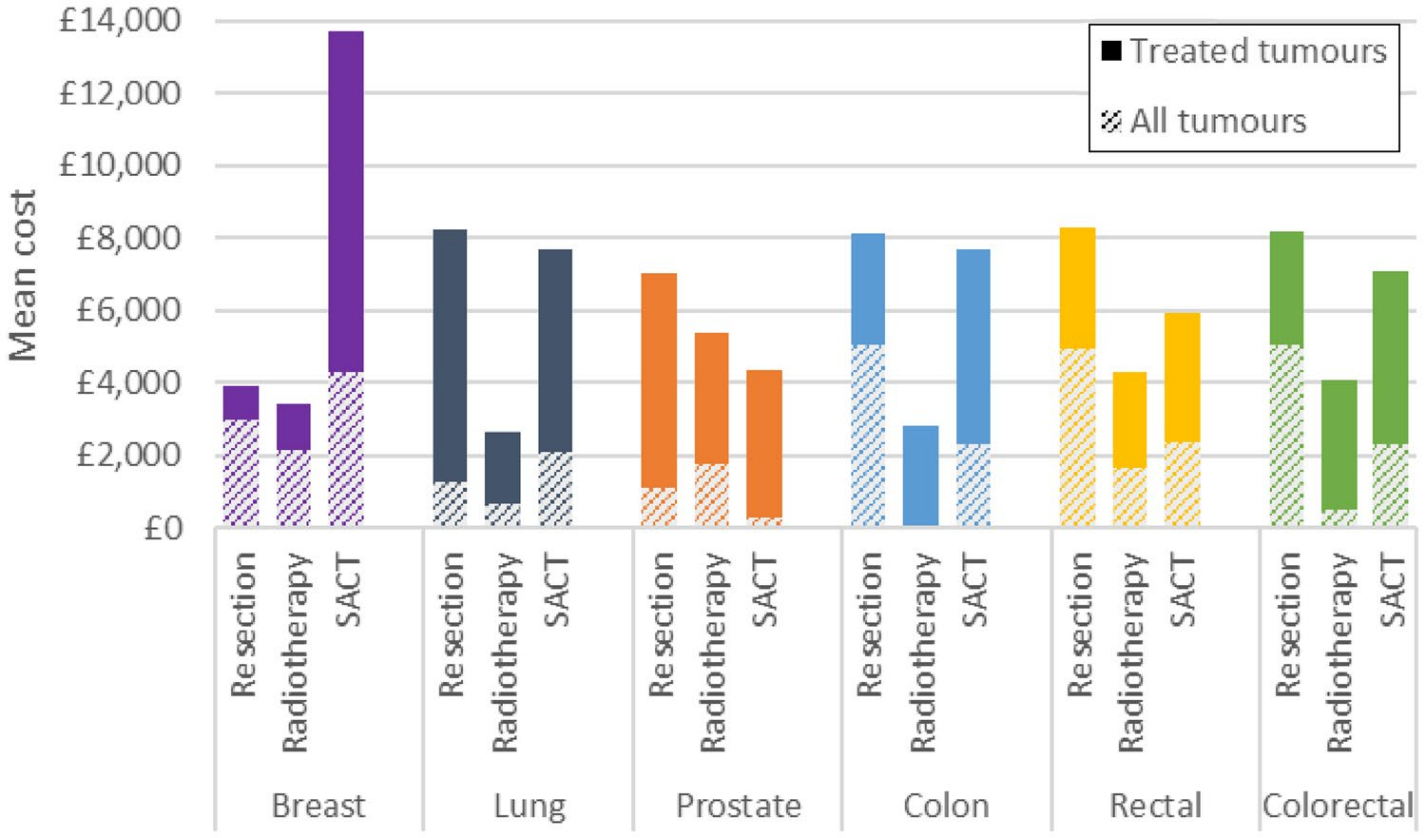
Mean cost of treatment by cancer and treatment modality. Solid bars show the incremental mean cost of treatment for treated tumours compared to the mean cost for all tumours. Treated tumours refers to tumours treated with the treatment modality of interest (alone or in combination with another modality). All tumours refers to all tumours in the analysis cohort regardless of whether they received costed treatment or not

For treated tumours, radiotherapy was typically the least costly treatment and resection the most costly (Fig. 5). For breast cancer, SACT was more costly than resection or radiotherapy.

Across all tumours in the cohort, the average cost of treating breast cancer was £9,450; £4,054 for lung cancer; £3,166 for prostate cancer; £7,437 for colon cancer and £8,988 for rectal cancer. Colon and rectal cancers combined had a mean total cost of £7,865. Within this total cost, patients could have a variety of treatment “pathways”, receiving any treatment modality alone or in combination with another modality or none of the included treatments. The mean cost among patients receiving each possible combination of the three treatments is presented in Online Resource 3.

The mean total cost of treatment decreased with increasing age and increasing number of comorbidities (Online Resource 4). There was also variation in mean total cost by gender, ethnicity and deprivation for certain cancers. Only lung and prostate cancers had a gradient of decreasing mean cost with increasing deprivation; colon and rectal cancers showed a similar trend when expanding to the full cohort. Other cancers showed no gradient or slightly increased costs with higher deprivation. The white ethnic group generally had lower mean total costs than other ethnic groups and males had a lower mean cost for lung cancer than females but higher costs for colon and rectal cancer.

## Discussion

This analysis calculated the costs of initial courses of resection, radiotherapy and SACT by stage at diagnosis, focusing on 455,789 tumours across five large cancer sites, reflecting the ‘real-world’ treatment received by patients in England. To our knowledge, this is the first analysis to include recent treatment costs by stage for cancer sites with the highest incidence in the UK. Our results indicate that treated breast, colon and rectal cancers had increasing mean total costs with later stage at diagnosis, while costs for treated lung and prostate cancers had lower costs at stages 1 and 4 compared to stages 2 and 3.

Variation was also identified in the mean cost by stage for each treatment modality individually. Limited variation in mean cost among treated tumours in stages 1–3 was observed within radiotherapy treatment, and also within SACT; radiotherapy costs decreased in stage 4, while SACT costs increased. The lower costs for stage 4 radiotherapy across cancer sites may largely be driven by palliative radiotherapy usage, which often consists of shorter courses of treatment than curative radiotherapy [29]. The cost of SACT was highest at stage 4 for all sites except prostate; the increasing use and availability of targeted therapies for later stage cancer [30] is likely to further increase this cost differential in the future. Since the study dates, more SACT options are now available which may further increase the cost of treatment at later stages.

Cost patterns differed when expanding the analysis to include all tumours in the cohort. The mean total cost of treatment generally increased from stages 1 to 3 and decreased at stage 4 for lung, prostate, colon and rectal cancers, suggesting that the cost is largely influenced by low proportions of patients having treatment at this stage. There are several reasons why fewer patients receive treatment in stage 4 and therefore why the overall cost of treatment might be lower at stage 4. Firstly, most curative treatments are ineffective for stage 4 cancers. Secondly, stage 4 patients may be less likely to receive treatment due to patient choice or being more likely to be unsuitable for treatment as they may have worse performance status, be frailer or have received prior treatment that may preclude subsequent treatment options. Thirdly, among patients who do have treatment, stage 4 patients may be less likely to tolerate or survive a full course of treatment, resulting in shortened and modified treatment schedules, and hence lower treatment costs. Finally, a higher proportion of patients receiving treatment at stage 4 may be taking part in a clinical trial for palliative SACT and hence excluded from our cohort [31].

Unlike the other sites, the mean total cost for breast cancer increased steadily from stages 1 to 4, even when including all tumours in the cohort; this is due to the high cost of SACT at later stages. Treatment costs for patients with breast cancer who were treated with SACT only were 6 to 10 times higher than when patients were treated with resection only or radiotherapy only, respectively, and were 1.4 to 4 times higher than when treated with any combination of treatment modalities. Patients diagnosed at stage 4 breast cancer are more likely to be treated with SACT alone compared to patients diagnosed at earlier stages [32], contributing to the lower overall costs for patients diagnosed with early-stage breast cancer. Diagnosing and treating breast cancer earlier can reduce the need for costly targeted therapies at later stages.

There was also variation in cost identified for demographic variables, including age, gender, ethnicity, deprivation and comorbidities. These findings are in line with previous literature that generally indicates decreasing cost of treatment with increasing age, higher levels of deprivation and more comorbidities [13–15, 33], likely due to frailty and lack of perceived suitability for treatment, resulting in reduced access to or uptake of treatment for these groups. This also potentially highlights areas where improvements could be made to ensure that all patients receive appropriate treatment. However, this analysis presents unadjusted results and so differing distributions of the other demographic variables may contribute to these findings. Further analysis could explore the independent contribution of all these variables to costs.

Previous work has found that breast, lung and colorectal cancers have lower costs at earlier stages of diagnosis [14, 19, 34, 35]. One study reported that in the year following diagnosis, patients aged 18–64 years diagnosed with breast cancer at stage 3 or 4 had £2,600 greater total cost of care than those diagnosed at stages 1 or 2, and those diagnosed with late-stage colorectal cancer had £4,300 greater total cost of care than early-stage [14]. Lower costs associated with earlier diagnosis were also sustained over a period of nine years post-diagnosis for breast and colorectal cancer. A systematic review of treatment costs of breast cancer found that mean treatment costs are 95% and 109% higher in stages 3 and 4, respectively, compared to stage 1 [19]. Analysis of cancer-attributable costs (direct cost of cancer treatment and other healthcare costs during treatment) for patients with lung cancer found that costs were higher in patients diagnosed at stage 3 or 4 compared to earlier stages [34], while another study found that in early stages, the main costs are incurred by surgery, while in later stages, radiotherapy, SACT and supportive care are more costly [35]. Previous analysis has found similar results to the current analysis for prostate cancer, with mean direct treatment costs increasing from stages 1 to 3 and decreasing at stage 4 [36]. Findings from previous research and the current study provide further evidence that there is a trend of lower costs in patients with breast, lung or colorectal cancer at earlier stages of diagnosis, and the cost benefit of diagnosing cancer earlier is sustained over time.

### Strengths and limitations

This study uses high quality cancer registration data in England linked with administrative hospital data collection for surgery, and specialised datasets for radiotherapy and SACT. This produces a large cohort of patients with a high proportion of staging data combined with treatment data assigned wherever captured. The costs have therefore been able to be applied dependent on the specific treatment received for each patient at each stage. This enables a straightforward estimate of the initial costs of treatment alone.

There are several limitations of this approach that should be considered when interpreting the results. Firstly, a substantial proportion of the cohort (33.6% of tumours) had no treatment with any of the three included modalities and were assigned a cost of £0. While some patients may genuinely receive no surgery, radiotherapy or SACT treatment, others may have had treatment that fell outside of our inclusion criteria, such as endocrine therapy or surgical treatment not aiming to remove the primary tumour. A small proportion may have received treatment privately, but this is believed to be minimal. The proportion receiving no treatment varied between cancer sites (ranging from 15.2% for breast cancer to 47.9% for prostate cancer), reflecting the different approaches to treatment such as watchful waiting and active surveillance for prostate cancer.

Secondly, there is the potential for overestimation of costs via the inclusion of treatment for previous or subsequent tumours to the diagnosis of interest, although this is expected to be minimal. Only 5% of people are diagnosed with two or more primary tumours in their lifetime [37] with the likelihood higher for those with good survival outcomes such as breast cancer. Excluding tumours with another diagnosis within 18 months, the limited timeframes for treatment inclusion and the use of site-specific OPCS codes were used to minimise this possibility. A further possible source of cost overestimation comes from the inclusion of endoscopic procedures for colon and rectal cancer at stage 1. While there is the potential for these procedures to fully resect early-stage tumours which resulted in their inclusion, it is also likely that this has resulted in the inclusion of some purely diagnostic procedures, with the cost of diagnosis not included for other sites and stages.

The timeframe within which treatment was included could also be a limitation for some cancer sites. This timeframe represented an ‘initial’ timeframe within which the start of primary treatment was likely to be captured. However, it is possible that these initial timeframes capture only a proportion of the whole treatment course, especially if the patient receives multiple sequential treatments, and that this proportion varies by stage [13] which could lead to a different relationship between mean cost and stage during the initial treatment period to that seen for the full cost of treatment. For example, watchful waiting or active surveillance is recommended for some patients with prostate cancer; if these patients eventually receive treatment but towards the end or outside of the post-diagnosis timeframe considered in this analysis, the initial treatment costs will be underestimated. Additionally, improvements in chemotherapy drugs have led to better survival for late-stage breast cancer patients receiving palliative treatment which means these patients receiving chemotherapy over a longer time period, and longer than captured by the timeframes used for this study. This could lead to greater costs for stage 4 breast cancer than those estimated here. Future costings analysis could potentially look at including treatment beyond this initial timeframe to provide a longer-term view of costs. This could be achieved similarly to the period approach in survival analysis by calculating the costs for treatment activity in a particular year but looking at cohorts in each subsequent year following diagnosis [14].

Another limitation is around the granularity of costs that we were able to apply. It was not possible to identify the setting where treatment was delivered, and so this analysis used the total unit cost of treatment, but the reference costs data has mean costs stratified by setting such as elective inpatient or day case and additional costs for length of stay over a set cut-off point for each HRG code. The setting and length of stay may also vary by stage at diagnosis and patient characteristics and so the use of total unit cost may mask some variation in costs. When relevant data is available in the Patient Level Information and Costing System (PLICS) dataset [38], future work could use this, as costs are collected at a patient level, removing the need to use mean costs. However, PLICS is not currently used to collect costs for radiotherapy and systemic anti-cancer treatment, and so was not suitable for the current analysis.

Some of the methodological limitations could lead to an underestimation of the cost at stage 4 relative to stages 1–3, such as the inclusion of only resection used to remove the primary tumour (thereby excluding any other surgical procedures undertaken, including those to alleviate symptoms), the exclusion of hormonal therapy and the use of an overall unit cost rather than cost stratified by setting and length of stay adjustments. The complexity of including a wider range of surgical procedures and hospital attendances while addressing the possibility of hospital attendances not being relevant to a cancer diagnosis could be addressed in future costings work by taking an approach analogous to net survival analysis, where the costs for all treatment received by a cancer population (such as all OPCS codes recorded in HES) are compared to the treatment costs for a general matched population in the same time period.

For prostate cancer specifically, treatment costs at stage 4 (and potentially for some stage 3) are likely underestimated due to the exclusion of hormonal therapy in the analysis. Hormonal therapy is known to be underreported in SACT, as some hormonal therapy is delivered outside an oncology environment such as in primary care, which is not fully captured in the SACT dataset [17]. This analysis focused on common treatment modalities (surgery, radiotherapy and chemotherapy) across the cancer sites of interest, and so hormonal therapy was not a focus of this work. The available literature points to a lack of data on hormonal therapy costs in prostate cancer in the England or UK context; further work is required to estimate these costs. It is unlikely that the inclusion of hormonal therapy for other cancer sites, including breast cancer, will impact the findings from this analysis, as the predominant usage of hormonal therapy for stage 4 breast cancer would increase the costs at this stage but not alter the relationship already seen between increasing cost with increasing stage.

Finally, our analysis did not capture all costs related to initial treatment for cancer, including the cost of treatment-related adverse events, such as hospital admissions or supportive medication, or follow-up. This analysis also did not include the costs of critical care or intensive care unit beds, which add to the cost of surgical treatment. Recovery times in intensive care or high dependency units will vary by procedures undertaken and patient factors and will differ by cancer site, however assigning a cost to this is related to the recovery of treatment rather than the actual treatment itself so has not been included. There are further costs associated with a cancer diagnosis and treatment that were not explored here. These include costs to the health system such as diagnostics, and any further supportive care required (subsequent surgery, physiotherapy, mental health support, etc.). Additionally, wider societal costs, such as direct costs to the patient through reduction in income and transport costs for attending appointments [39], were not included. Further, indirect costs to the UK economy through premature mortality and to patients through time off work and unpaid care by friends and family [40] were not considered; indirect costs are estimated to account for over 60% of all costs in the UK [41]. These costs and their impact are also likely to vary by cancer site, stage at diagnosis, treatment received and individual patient circumstances and are therefore also important to consider in the context of the economic implications of earlier cancer diagnosis. As this study used cancer registry and cost data from England, findings may not be generalisable to other countries with different healthcare systems; further research should be considered in other nations as this type of analysis is informative for planning cancer services and developing policy.

## Conclusions

This analysis is the first to estimate initial treatment costs by stage based on observed treatment-related data, focusing on the cancer sites with the highest incidence in the UK. Mean total cost increased with later stage at diagnosis for treated breast, colon and rectal cancers, while costs for treated lung and prostate cancers were lower at stages 1 and 4 compared to stages 2 and 3. In general, surgery and SACT were the costliest treatments. These findings also identified potential variation in the cost of initial treatment by demographic variables, including age, gender, ethnicity, deprivation and comorbidities, highlighting possible areas where improvements could be made to ensure that all patients receive appropriate treatment. Future cost of treatment analysis would benefit from using longer treatment inclusion timeframes and tumour-level matching for treatments. In addition, future research could include additional costs to the health system, including diagnostics, hospital stays and additional medications, for a holistic picture of the costs of initial treatment. Diagnosing cancers earlier has the potential for reducing the long-term burden on the NHS and yielding large cost savings in the initial treatment period.

### Supplementary Information

Below is the link to the electronic supplementary material.

Supplementary file1 (PDF 243 KB)

Supplementary file2 (PDF 121 KB)

Supplementary file3 (PDF 120 KB)

Supplementary file4 (DOCX 45 KB)

## Data Availability

All data analysed during this study are available from NHS Digital’s National Cancer Registration and Analysis Service, Admitted Patient Care Hospital Episode Statistics, Radiotherapy Dataset, Systemic Anti-Cancer Therapy dataset and NHS Improvement’s Archived Reference Costs. However, restrictions apply to the availability of these data.
