# Supplementary material for: Estimating surgery, radiotherapy and systemic anti-cancer therapy treatment costs for cancer patients by stage at diagnosis
Source: Eur J Health Econ. 2023 Sep 1;25(5):763–74. doi: 10.1007/s10198-023-01623-5 (PMC11192664; doi:10.1007/s10198-023-01623-5)
Supplement: Supplementary file 1 — Supplementary file1 (PDF 243 KB) [file 10198_2023_1623_MOESM1_ESM.pdf]

Estimating surgery, radiotherapy and systemic anti-cancer therapy treatment costs for cancer patients by stage at diagnosis

The European Journal of Health Economics

Authors: Lorna Wills, Diana Nagarwalla*, Clare Pearson, Sean McPhail, Rose Hinchliffe, Ben Sharpless, Fahmina Fardus-Reid, Lyndsy Ambler, Samantha Harrison, Jon Shelton

*** Corresponding author**
Diana Nagarwalla
Cancer Research UK, 2 Redman Place, London, E20 1JQ, UK
Diana.Nagarwalla@cancer.org.uk

ONLINE RESOURCE 1 – METHODOLOGY

Table A1 Definition of treatment start, how treatment is defined and timeframes for including treatment

	**Tumour resections**	**Radiotherapy**	**Systemic anti-cancer therapy**
**How is treatment start defined**	Date of admission within timeframe	Treatment start date (for episode) within timeframe	Start date of regimen within timeframe
**How is treatment defined**	Site- and stage-specific OPCS procedure codes that would be used to remove the primary tumour	All recorded within RTDS with a radiotherapy delivery and/or preparation OPCS code, including brachytherapy (although there are known data completeness issues with brachytherapy recording)	All recorded within SACT with a C-code primary diagnosis with the exclusion of solely hormone or supportive therapy
	**Days included as post-diagnostic timeframe**		
**Breast**	365	365	365
**Lung**	183	183	183
**Prostate**	456	365	365
**Colon**	183	365	365
**Rectal**	365	365	365

Abbreviations: OPCS – Operating Procedure Codes Supplement; RTDS – Radiotherapy Dataset; SACT – Systemic Anti-Cancer Therapy

Matching treatment data to costings data and calculating costs per tumour

For resections we used the Secondary Uses Services (SUS)-generated Healthcare Resource Group (HRG) field within Admitted Patient Care Hospital Episode Statistics (HES) to match episodes of care to the reference costs data. Where a SUS HRG code did not appear in the 2017/2018 reference costs data, HRG codes present in historic reference costs data were matched, where possible, to equivalent 2017/2018 HRG codes based on the description of the procedure the code related to. For certain historic HRG codes, a match could be found to multiple 2017/2018 codes, in which case an average cost for all relevant matched 2017/2018 HRG codes was used. A small number of SUS HRG codes (nine out of 443; 0.7% episodes) were unable to be matched to the 2017/2018 reference costs data but were present in the 2016/2017 reference costs data and these costs were used as needed. Where it was not possible to match a SUS HRG code to any cost using the methods above, a cost of £0 was assigned to that episode (see **Figure A1**).

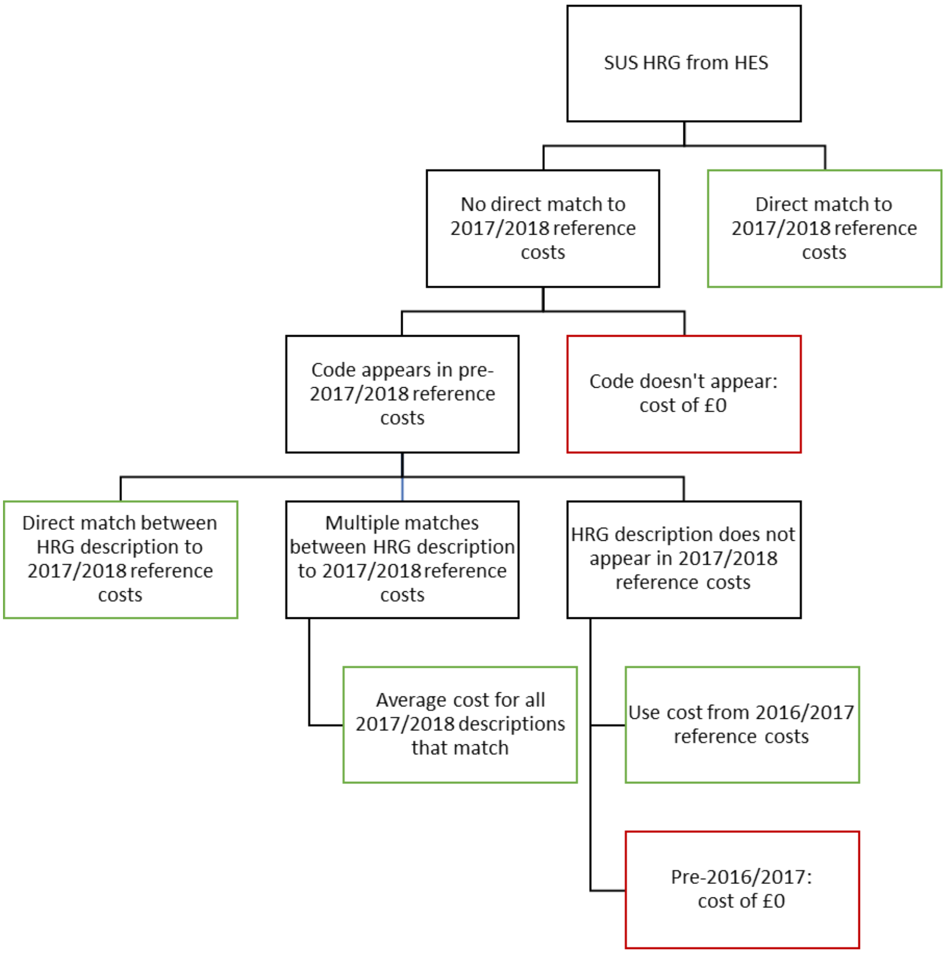

Fig A1 Matching resective surgery HRG codes to costs

Abbreviations: HES – Hospital Episode Statistics; HRG – Healthcare Resource Group; SUS – Secondary Uses Services

For radiotherapy, the ‘primaryprocedureopcs’ field was used to assign delivery and preparation HRG codes to each attendance using the rules described in the HRG grouper tool (33) for generating HRG codes from Operating Procedure Codes Supplement (OPCS) codes. Delivery and preparation OPCS were summarised at the attendance level to produce overall HRG codes. Where a patient received multiple radiotherapy doses on a single day (hyperfractionation), each dose was treated as a separate attendance. Limitations in recording within the Radiotherapy Dataset pre-April 2016 meant that fractions delivered on the same day were unable to be distinguished and so hyperfractionation was unable to be factored into costs for radiotherapy delivered pre-April 2016. As preparation codes relate to a treatment course rather than each individual attendance, only a small portion of attendances had a preparation code.[1] It was assumed that each attendance would only have a single delivery code and so, where multiple codes were present for an attendance, the average cost of these codes was taken. It was also assumed that multiple preparation codes could be assigned to an attendance and so, where multiple codes were present, the total cost was used.

Procurement and delivery OPCS codes were assigned to each cycle of systemic anti-cancer therapy (SACT), with OPCS codes directly corresponding to HRG codes for SACT. There was no single variable that could be used to assign OPCS codes to all cycles of SACT and so OPCS codes were assigned in a hierarchical way (see **Figure A2**):

1. The mapped_regimen field was matched to the 2017/2018 National Tariff Chemotherapy Regimens List[2] which lists the OPCS codes associated with a particular named regimen;
2. Where no direct match to the National Tariff Chemotherapy Regimens List was possible, but the mapped_regimen name appeared on the list with multiple different options (e.g., length of cycle, delivery methods, amount of drug given), then the SACT OPCS code, where present, was used;
3. If no match could be made to the National Tariff Chemotherapy Regimens List and the SACT OPCS code was X708/X729 (procurement/delivery for regimens not on the National List) then this code was used;
4. If none of the above criteria were met, HES OPCS codes matched on the patientid and start date of cycle to the HES based appointment were used.

In some cases, it was not possible to assign an OPCS/HRG code to an individual cycle of treatment via the above approaches, so costs were calculated and applied directly to these cases as follows:

5. Average cost generated from applying costs to SACT OPCS codes recorded for each individual named regimen and used if mapped_regimen name appeared multiple times on the National Tariff Chemotherapy Regimens List or the percentage of cycles with a missing SACT OPCS code was 50% or lower;
6. Otherwise the cost for procuring/delivering regimens not on the National List was used.

Alongside the cost of procuring and delivering a cycle of chemotherapy, which is incurred at the start of a cycle, there is also a cost for delivering subsequent elements of a cycle. The number of additional attendances within a cycle was calculated from the number of administration dates recorded for a cycle and the cost for delivering subsequent elements of a cycle added to the total cost. Note that the SACT dataset consists of a current and historic dataset which must be combined to give all SACT data for our time-period of interest. There is some cross-over in this dataset and the method used to combine these two datasets is likely to have resulted in a slight undercount of additional attendances.

For the sensitivity analysis of assigning costs to SACT due to the uncertainty around the costing process, step 5 was removed from the above.

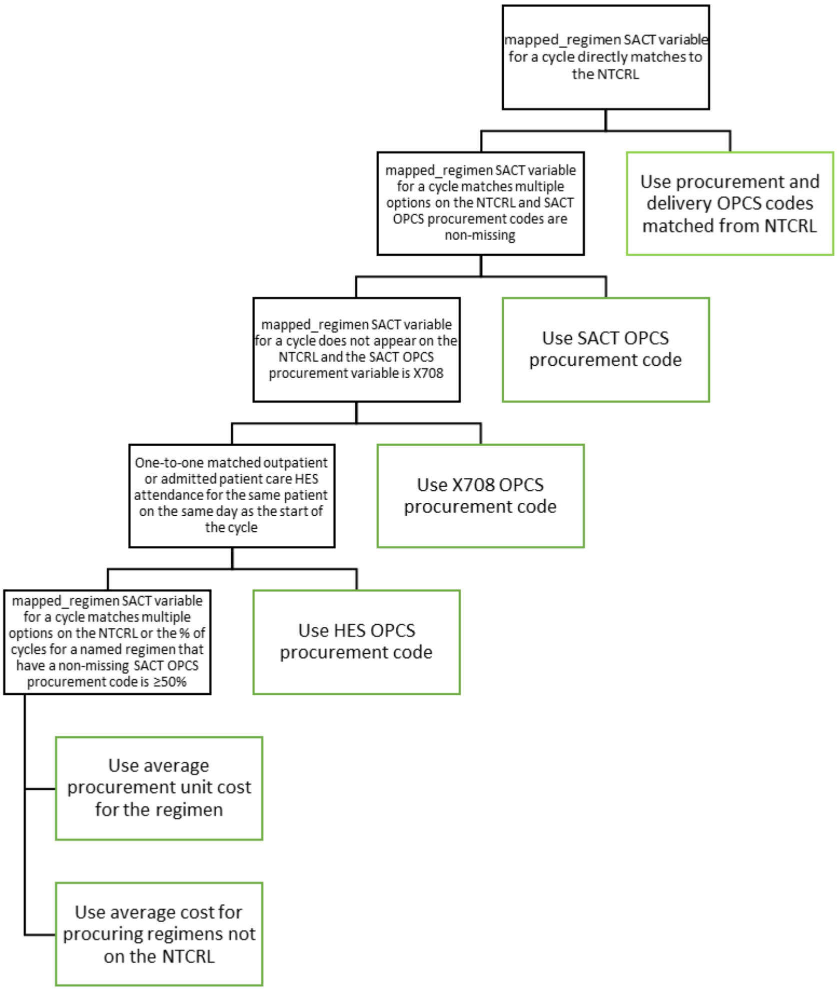

Fig A2 Matching systemic anti-cancer therapy cycles to an OPCS code or average cost

Abbreviations: HES – Hospital Episode Statistics; NTCRL – National Tariff Chemotherapy Regimens List; OPCS – Operating Procedure Codes Supplement; SACT – systemic anti-cancer therapy

Summary details of the costing process

Of 223,376 surgical episodes, 677 (0.3%) could not be costed. The proportion of episodes costed by site and stage was over 99% for all site and stage combinations except for stage 1 rectal cancer with 98.5%.

4,143 out of 169,777 radiotherapy episodes (2.4%) had no preparation code. Cancers diagnosed at stage 1 generally had a lower proportion of episodes without a preparation code, except for stage 1 lung cancer which had the highest proportion without a preparation code for any site/stage combination (9.8% of episodes). 5,339 out of 2,673,611 attendances (0.2%) had multiple delivery

flags. This varied by site and stage with lung cancer having the highest proportion of attendances with multiple delivery flags (for each stage), although this was below 0.8% for all site and stage combinations.

For SACT, 69.9% of cycles were able to be matched to the National Tariff Chemotherapy Regimens List. 77.5% of cycles had an OPCS procurement code recorded within the SACT database and 78.7% had an OPCS delivery code. 74.4% of cycles were able to be uniquely matched to an OPCS procurement code from HES and 76.8% to an OPCS delivery code. Combining these three variables together resulted in 95.3% of cycles having a procurement code and 95.7% having a delivery code. For the remaining cycles, a mean cost per regimen was calculated and applied for those regimens which matched to multiple items on the National Tariff Chemotherapy Regimens list or with 50% or fewer SACT OPCS codes missing which resulted in 98.4% of cycles having a procurement cost and 99.2% having a delivery cost.

There was variation in these numbers by site and stage. The proportion matching to the National Tariff Chemotherapy Regimens List was lower for breast and prostate than lung, colon and rectal cancer; stage 4 breast cancer had the lowest proportion of cycles with a SACT OPCS procurement or delivery code (55.2% and 57.1%, respectively). Stage 1 prostate cancer had the lowest proportion of cycles that were able to be assigned a procurement/delivery cost via any method (95.7% for both) and hence the highest proportion allocated the cost for procuring/delivering regimens not on the National List.

Calculating cost per tumour

$$Total\ cost\ of\ resection = \sum(cost\ per\ episode)$$

$$Total\ cost\ of\ radiotherapy = \sum(average\ delivery\ cost\ per\ attendance + total\ preparation\ cost\ per\ attendance)$$

$$Total\ cost\ of\ systemic\ anti-cancer\ therapy = \sum(procurement\ cost\ per\ cycle + delivery\ cost\ per\ cycle + number\ of\ subsequent\ attendances\ for\ a\ cycle * cost\ of\ delivering\ subsequent\ elements\ of\ a\ cycle)$$

$$Overall\ cost\ of\ treatment = total\ cost\ of\ resection + total\ cost\ of\ radiotherapy + total\ cost\ of\ systemic\ anti-cancer\ therapy$$

1. NHS. 2020/21 National Cost Collection guidance Volume 3: National Cost Collection - acute, mental health and improving access to psychological therapies (IAPT). https://www.england.nhs.uk/wp-content/uploads/2021/03/ACG-Vol-3-2021-Final.pdf (2021). Accessed 7 June 2022
2. Clinical Classifications Service. National Tariff Chemotherapy Regimens List 2017-2018 Version 1.0.
