# Supplementary material for: Estimating surgery, radiotherapy and systemic anti-cancer therapy treatment costs for cancer patients by stage at diagnosis
Source: Eur J Health Econ. 2023 Sep 1;25(5):763–74. doi: 10.1007/s10198-023-01623-5 (PMC11192664; doi:10.1007/s10198-023-01623-5)
Supplement: Supplementary file 2 — Supplementary file2 (PDF 121 KB) [file 10198_2023_1623_MOESM2_ESM.pdf]

Estimating surgery, radiotherapy and systemic anti-cancer therapy treatment costs for cancer patients by stage at diagnosis

The European Journal of Health Economics

Authors: Lorna Wills, Diana Nagarwalla*, Clare Pearson, Sean McPhail, Rose Hinchliffe, Ben Sharpless, Fahmina Fardus-Reid, Lyndsy Ambler, Samantha Harrison, Jon Shelton

*** Corresponding author**
Diana Nagarwalla
Cancer Research UK, 2 Redman Place, London, E20 1JQ, UK
Diana.Nagarwalla@cancer.org.uk

ONLINE RESOURCE 2 – NUMBER OF TUMOURS TREATED WITH EACH TREATMENT MODALITY BY SITE AND STAGE

Cancer site	Stage	Number of tumours	Number having any treatment (%)	Number having resective surgery (%)	Number having radiotherapy (%)	Number having systemic anti-cancer therapy (%)
**Breast**	All	125,294	106,230 (84.8%)	96,183 (76.8%)	79,877 (63.8%)	39,445 (31.5%)
	1	51,801	47,104 (90.9%)	44,885 (86.6%)	35,592 (68.7%)	7,707 (14.9%)
	2	48,351	42,040 (86.9%)	38,948 (80.6%)	31,517 (65.2%)	20,270 (41.9%)
	3	10,548	9,503 (90.1%)	8,444 (80.1%)	8,200 (77.7%)	6,982 (66.2%)
	4	6,090	3,757 (61.7%)	957 (15.7%)	1,994 (32.7%)	2,710 (44.5%)
	Unknown	8,504	3,826 (45.0%)	2,949 (34.7%)	2,574 (30.3%)	1,776 (20.9%)
**Lung**	All	108,602	58,920 (54.3%)	16,390 (15.1%)	28,324 (26.1%)	29,967 (27.6%)
	1	18,985	13,861 (73.0%)	9,619 (50.7%)	4,298 (22.6%)	626 (3.3%)
	2	7,876	5,654 (71.8%)	3,527 (44.8%)	2,037 (25.9%)	2,101 (26.7%)
	3	21,286	13,878 (65.2%)	2,396 (11.3%)	8,198 (38.5%)	9,593 (45.1%)
	4	52,803	24,651 (46.7%)	541 (1.0%)	13,400 (25.4%)	17,325 (32.8%)
	Unknown	7,652	876 (11.4%)	307 (4.0%)	391 (5.1%)	322 (4.2%)
**Prostate**	All	125,609	65,491 (52.1%)	19,785 (15.8%)	41,766 (33.3%)	7,728 (6.2%)
	1	41,314	15,636 (37.8%)	4,293 (10.4%)	11,299 (27.3%)	180 (0.4%)
	2	19,754	13,552 (68.6%)	6,734 (34.1%)	6,909 (35.0%)	77 (0.4%)
	3	28,135	21,546 (76.6%)	7,314 (26.0%)	14,821 (52.7%)	520 (1.8%)
	4	23,688	11,929 (50.4%)	810 (3.4%)	6,728 (28.4%)	6,626 (28.0%)
	Unknown	12,718	2,828 (22.2%)	634 (5.0%)	2,009 (15.8%)	325 (2.6%)
**Colon**	All	69,713	49,933 (71.6%)	43,292 (62.1%)	2,030 (2.9%)	20,855 (29.9%)
	1	9,536	8,846 (92.8%)	8,834 (92.6%)	67 (0.7%)	157 (1.6%)
	2	17,435	15,264 (87.5%)	14,965 (85.8%)	265 (1.5%)	2,941 (16.9%)
	3	17,285	14,751 (85.3%)	13,769 (79.7%)	752 (4.4%)	9,538 (55.2%)
	4	17,935	9,561 (53.3%)	4,534 (25.3%)	804 (4.5%)	7,676 (42.8%)
	Unknown	7,522	1,511 (20.1%)	1,190 (15.8%)	142 (1.9%)	543 (7.2%)
**Rectal**	All	26,571	22,287 (83.9%)	15,942 (60.0%)	10,264 (38.6%)	10,658 (40.1%)
	1	6,184	6,009 (97.2%)	5,963 (96.4%)	1,159 (18.7%)	517 (8.4%)
	2	4,501	4,009 (89.1%)	3,176 (70.6%)	1,639 (36.4%)	1,291 (28.7%)
	3	8,779	8,123 (92.5%)	5,658 (64.4%)	5,225 (59.5%)	5,756 (65.6%)
	4	4,964	3,472 (69.9%)	820 (16.5%)	1,805 (36.4%)	2,813 (56.7%)
	Unknown	2,143	674 (31.5%)	325 (15.2%)	436 (20.3%)	281 (13.1%)
**Colorectal**	All	96,284	72,220 (75.0%)	59,234 (61.5%)	12,294 (12.8%)	31,513 (32.7%)
	1	15,720	14,855 (94.5%)	14,797 (94.1%)	1,226 (7.8%)	674 (4.3%)
	2	21,936	19,273 (87.9%)	18,141 (82.7%)	1,904 (8.7%)	4,232 (19.3%)
	3	26,064	22,874 (87.8%)	19,427 (74.5%)	5,977 (22.9%)	15,294 (58.7%)
	4	22,899	13,033 (56.9%)	5,354 (23.4%)	2,609 (11.4%)	10,489 (45.8%)
	Unknown	9,665	2,185 (22.6%)	1,515 (15.7%)	578 (6.0%)	824 (8.5%)
