# Supplementary material for: Estimating surgery, radiotherapy and systemic anti-cancer therapy treatment costs for cancer patients by stage at diagnosis
Source: Eur J Health Econ. 2023 Sep 1;25(5):763–74. doi: 10.1007/s10198-023-01623-5 (PMC11192664; doi:10.1007/s10198-023-01623-5)
Supplement: Supplementary file 3 — Supplementary file3 (PDF 120 KB) [file 10198_2023_1623_MOESM3_ESM.pdf]

Estimating surgery, radiotherapy and systemic anti-cancer therapy treatment costs for cancer patients by stage at diagnosis

The European Journal of Health Economics

Authors: Lorna Wills, Diana Nagarwalla*, Clare Pearson, Sean McPhail, Rose Hinchliffe, Ben Sharpless, Fahmina Fardus-Reid, Lyndsy Ambler, Samantha Harrison, Jon Shelton

*** Corresponding author**
Diana Nagarwalla
Cancer Research UK, 2 Redman Place, London, E20 1JQ, UK
Diana.Nagarwalla@cancer.org.uk

ONLINE RESOURCE 3 – MEAN COST OF TREATMENT COMBINATIONS BY SITE

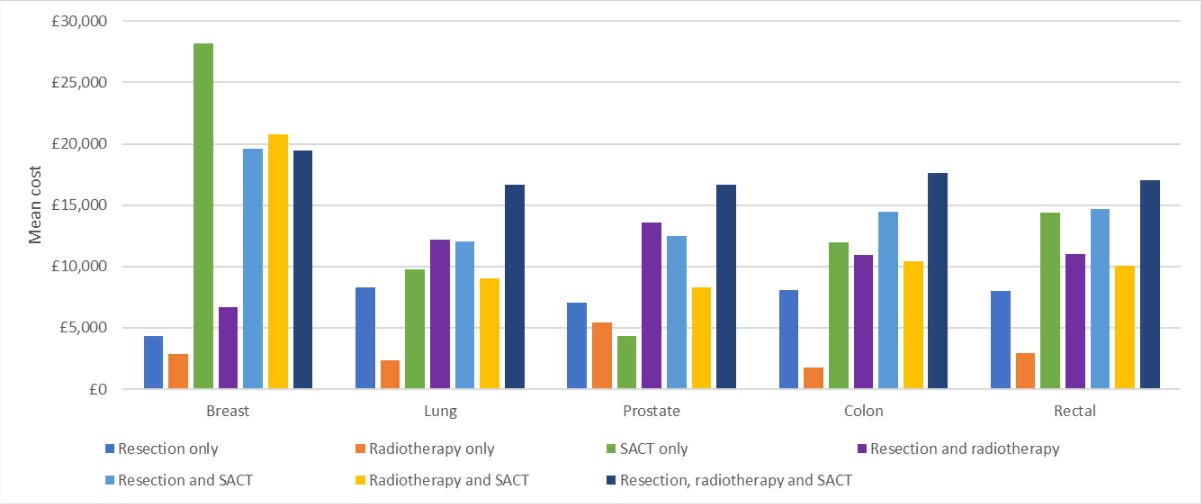

Note: The total costs presented here for resection, radiotherapy and SACT only differ from total costs by treatment modality displayed in Figure 5. Costs in Figure 5 are for patients who have had the treatment modality regardless of whether or not they received the other two treatment modalities. The figure here presents costs for patients who were treated with *only* one treatment modality and not the others.
