# Supplementary material for: Estimating surgery, radiotherapy and systemic anti-cancer therapy treatment costs for cancer patients by stage at diagnosis
Source: Eur J Health Econ. 2023 Sep 1;25(5):763–74. doi: 10.1007/s10198-023-01623-5 (PMC11192664; doi:10.1007/s10198-023-01623-5)
Supplement: Supplementary file 4 — Supplementary file4 (DOCX 45 KB) [file 10198_2023_1623_MOESM4_ESM.docx]

**Estimating surgery, radiotherapy and systemic anti-cancer therapy treatment costs for cancer patients by stage at diagnosis**

The European Journal of Health Economics

**Authors:** Lorna Wills, Diana Nagarwalla*, Clare Pearson, Sean McPhail, Rose Hinchliffe, Ben Sharpless, Fahmina Fardus-Reid, Lyndsy Ambler, Samantha Harrison, Jon Shelton

*** Corresponding author**

Diana Nagarwalla

Cancer Research UK, 2 Redman Place, London, E20 1JQ, UK

[Diana.Nagarwalla@cancer.org.uk](mailto:Diana.Nagarwalla@cancer.org.uk)

**Online Resource 4 – Mean overall cost by tumour site and demographic variable**

**Table 1 Mean overall cost for breast, lung and prostate cancers by demographic variable**

|  | **Breast** | | | **Lung** | | | **Prostate** | | |
| --- | --- | --- | --- | --- | --- | --- | --- | --- | --- |
|  | **Mean overall cost, treated tumours** | **% receiving treatment** | **Mean overall cost, full cohort** | **Mean overall cost, treated tumours** | **% receiving treatment** | **Mean overall cost, full cohort** | **Mean overall cost, treated tumours** | **% receiving treatment** | **Mean overall cost, full cohort** |
| **Total** | £11,146 | 84.8% | £9,450 | £7,473 | 54.3% | £4,054 | £6,073 | 52.1% | £3,166 |
| **Age** | | | | | | | | | |
| <45 | £16,336 | 92.4% | £15,089 | £11,222 | 79.3% | £8,903 | £7,057 | 50.0% | £3,528 |
| 45-54 | £13,078 | 93.4% | £12,210 | £9,348 | 76.8% | £7,182 | £6,853 | 59.7% | £4,088 |
| 55-64 | £11,379 | 94.1% | £10,706 | £8,359 | 72.2% | £6,032 | £6,570 | 61.5% | £4,037 |
| 65-74 | £9,600 | 91.9% | £8,826 | £7,613 | 63.7% | £4,853 | £6,130 | 62.6% | £3,834 |
| 75-84 | £7,642 | 73.2% | £5,591 | £6,421 | 46.2% | £2,966 | £5,325 | 40.7% | £2,169 |
| 85+ | £4,772 | 28.5% | £1,361 | £4,309 | 17.6% | £758 | £1,921 | 6.0% | £116 |
| **Comorbidity** | | | | | | | | | |
| 0 | £11,345 | 87.3% | £9,902 | £7,885 | 57.8% | £4,556 | £6,122 | 54.8% | £3,353 |
| 1 | £10,107 | 76.5% | £7,729 | £6,817 | 53.8% | £3,667 | £5,829 | 45.4% | £2,647 |
| 2 | £8,931 | 65.2% | £5,822 | £6,218 | 46.7% | £2,904 | £5,728 | 38.6% | £2,213 |
| 3+ | £7,466 | 49.2% | £3,673 | £5,731 | 36.6% | £2,096 | £5,417 | 26.0% | £1,407 |
| **Deprivation** | | | | | | | | | |
| 1 – Least deprived | £10,981 | 84.6% | £9,286 | £8,101 | 56.7% | £4,591 | £6,077 | 53.9% | £3,274 |
| 2 | £10,962 | 85.1% | £9,331 | £7,841 | 55.0% | £4,315 | £6,099 | 52.8% | £3,218 |
| 3 | £10,931 | 85.0% | £9,287 | £7,515 | 54.5% | £4,094 | £6,115 | 51.9% | £3,175 |
| 4 | £11,422 | 84.7% | £9,678 | £7,264 | 53.1% | £3,860 | £6,052 | 51.0% | £3,085 |
| 5 – Most deprived | £11,644 | 84.4% | £9,832 | £6,974 | 53.1% | £3,706 | £5,965 | 49.4% | £2,949 |
| **Ethnicity** | | | | | | | | | |
| Asian | £12,315 | 89.5% | £11,019 | £10,037 | 59.4% | £5,963 | £6,053 | 54.4% | £3,291 |
| Black | £13,910 | 89.2% | £12,408 | £9,781 | 63.1% | £6,170 | £6,495 | 50.8% | £3,299 |
| White | £11,040 | 85.5% | £9,441 | £7,388 | 55.0% | £4,062 | £6,051 | 52.7% | £3,187 |
| Mixed | £13,252 | 91.5% | £12,121 | £8,106 | 60.9% | £4,939 | £6,510 | 54.4% | £3,539 |
| Other | £13,754 | 88.2% | £12,130 | £9,054 | 62.6% | £5,664 | £6,214 | 54.6% | £3,392 |
| Not stated or known | £9,430 | 66.1% | £6,231 | £6,749 | 31.2% | £2,108 | £6,098 | 45.0% | £2,747 |
| **Gender** | | | | | | | | | |
| Female | £11,146 | 84.8% | £9,450 | £7,815 | 54.9% | £4,287 | – | – | – |
| Male | – | – | – | £7,156 | 53.7% | £3,843 | £6,073 | 52.1% | £3,166 |
| **Stage** | | | | | | | | | |
| 1 | £8,266 | 90.9% | £7,517 | £7,018 | 73.0% | £5,124 | £5,616 | 37.8% | £2,126 |
| 2 | £12,051 | 86.9% | £10,478 | £8,003 | 71.8% | £5,745 | £6,319 | 68.6% | £4,335 |
| 3 | £15,472 | 90.1% | £13,939 | £7,968 | 65.2% | £5,195 | £6,763 | 76.6% | £5,179 |
| 4 | £25,049 | 61.7% | £15,453 | £7,345 | 46.7% | £3,429 | £5,220 | 50.4% | £2,629 |
| Unknown | £12,261 | 45.0% | £5,516 | £6,973 | 11.4% | £798 | £5,749 | 22.2% | £1,278 |

Note: Treated tumours refers to tumours treated with at least one treatment modality of interest (alone or in combination with another modality). All tumours refers to all tumours in the analysis cohort regardless of whether they received costed treatment or not.

**Table 2 Mean overall cost for colon and rectal cancers by demographic variable**

|  | **Colon** | | | **Rectal** | | | **Colorectal** | | |
| --- | --- | --- | --- | --- | --- | --- | --- | --- | --- |
|  | **Mean overall cost, treated tumours** | **% receiving treatment** | **Mean overall cost, full cohort** | **Mean overall cost, treated tumours** | **% receiving treatment** | **Mean overall cost, full cohort** | **Mean overall cost, treated tumours** | **% receiving treatment** | **Mean overall cost, full cohort** |
| **Total** | £10,383 | 71.6% | £7,437 | £10,716 | 83.9% | £8,988 | £10,486 | 75.0% | £7,865 |
| **Age** | | | | | | | | | |
| <45 | £13,267 | 83.8% | £11,117 | £13,838 | 88.2% | £12,208 | £13,446 | 85.1% | £11,448 |
| 45-54 | £12,746 | 87.4% | £11,144 | £13,148 | 91.5% | £12,030 | £12,890 | 88.8% | £11,453 |
| 55-64 | £11,592 | 87.1% | £10,095 | £12,032 | 91.7% | £11,030 | £11,748 | 88.7% | £10,415 |
| 65-74 | £10,433 | 83.1% | £8,672 | £11,043 | 90.3% | £9,967 | £10,624 | 85.2% | £9,055 |
| 75-84 | £9,124 | 68.2% | £6,223 | £8,939 | 78.8% | £7,047 | £9,076 | 70.7% | £6,415 |
| 85+ | £7,874 | 34.7% | £2,730 | £4,932 | 53.3% | £2,631 | £7,057 | 38.4% | £2,711 |
| **Comorbidity** | | | | | | | | | |
| 0 | £10,630 | 75.5% | £8,026 | £11,065 | 85.9% | £9,501 | £10,767 | 78.5% | £8,452 |
| 1 | £9,539 | 66.0% | £6,291 | £9,337 | 78.1% | £7,294 | £9,484 | 68.9% | £6,532 |
| 2 | £9,227 | 56.7% | £5,236 | £8,956 | 75.2% | £6,734 | £9,151 | 60.9% | £5,577 |
| 3+ | £8,583 | 45.2% | £3,881 | £7,194 | 65.5% | £4,711 | £8,199 | 49.4% | £4,054 |
| **Deprivation** | | | | | | | | | |
| 1 – Least deprived | £10,429 | 74.1% | £7,729 | £10,600 | 85.3% | £9,041 | £10,481 | 77.2% | £8,091 |
| 2 | £10,201 | 73.7% | £7,521 | £10,725 | 85.3% | £9,146 | £10,359 | 76.9% | £7,962 |
| 3 | £10,247 | 70.7% | £7,240 | £10,649 | 84.3% | £8,975 | £10,375 | 74.5% | £7,729 |
| 4 | £10,530 | 70.3% | £7,402 | £10,679 | 82.8% | £8,843 | £10,576 | 73.8% | £7,802 |
| 5 – Most deprived | £10,610 | 68.0% | £7,210 | £11,013 | 80.6% | £8,875 | £10,734 | 71.4% | £7,663 |
| **Ethnicity** | | | | | | | | | |
| Asian | £10,483 | 78.8% | £8,261 | £11,414 | 86.7% | £9,891 | £10,816 | 81.4% | £8,808 |
| Black | £10,766 | 75.1% | £8,089 | £10,960 | 79.7% | £8,733 | £10,810 | 76.1% | £8,230 |
| White | £10,388 | 72.3% | £7,507 | £10,727 | 84.7% | £9,085 | £10,492 | 75.7% | £7,941 |
| Mixed | £11,485 | 79.9% | £9,179 | £11,046 | 84.8% | £9,372 | £11,351 | 81.4% | £9,235 |
| Other | £11,352 | 77.4% | £8,785 | £11,348 | 86.9% | £9,856 | £11,351 | 80.0% | £9,085 |
| Not stated or known | £9,664 | 54.5% | £5,268 | £9,714 | 68.5% | £6,654 | £9,680 | 58.4% | £5,650 |
| **Gender** | | | | | | | | | |
| Female | £10,064 | 69.2% | £6,967 | £10,154 | 81.1% | £8,234 | £10,087 | 71.9% | £7,251 |
| Male | £10,660 | 73.8% | £7,871 | £11,021 | 85.5% | £9,419 | £10,786 | 77.5% | £8,363 |
| **Stage** | | | | | | | | | |
| 1 | £6,718 | 92.8% | £6,232 | £7,387 | 97.2% | £7,178 | £6,989 | 94.5% | £6,604 |
| 2 | £9,138 | 87.5% | £8,000 | £10,360 | 89.1% | £9,228 | £9,392 | 87.9% | £8,252 |
| 3 | £11,431 | 85.3% | £9,755 | £12,305 | 92.5% | £11,385 | £11,741 | 87.8% | £10,304 |
| 4 | £14,209 | 53.3% | £7,575 | £13,438 | 69.9% | £9,399 | £14,004 | 56.9% | £7,970 |
| Unknown | £9,986 | 20.1% | £2,006 | £9,325 | 31.5% | £2,933 | £9,782 | 22.6% | £2,211 |

Note: Treated tumours refers to tumours treated with at least one treatment modality of interest (alone or in combination with another modality). All tumours refers to all tumours in the analysis cohort regardless of whether they received costed treatment or not.
